# ASO Author Reflections: Imaging the Metabolic Signature of Periprostatic Fat: Advancing Risk Stratification in Prostate Cancer

**DOI:** 10.1245/s10434-025-17644-8

**Published:** 2025-06-23

**Authors:** Chih-Yu Shen, Jhih-Kai Pan, Wen-Der Lin, Hui-Chuan Cheng, Che-Yuan Hu, Kuan-Yu Wu, Yung-Ming Kuo, Gia-Shing Shieh, Pei-Jung Lu

**Affiliations:** 1 Institute of Clinical Medicine, College of Medicine, National Cheng Kung University, Tainan, Taiwan; 2 Department of Urology, Tainan Hospital, Ministry of Health and Welfare, Tainan, Taiwan; 3 Department of Urology, National Cheng Kung University Hospital, College of Medicine, National Cheng Kung University, Tainan, Taiwan; 4 Department of Electronic Engineering, College of Electrical and Computer of Engineering, National Formosa University, Yunlin County, Taiwan

## Past

Prostate cancer (PCa) is marked by a significant degree of heterogeneity, presenting a challenge for precision therapy targeting the tumor microenvironment (TME). Traditional tools such as prostate biopsies and serum tumor markers have limitations in providing a comprehensive view of the disease. In contrast, magnetic resonance imaging (MRI) offers advantages including noninvasiveness, soft-tissue characterization, and the absence of ionizing radiation, making it promising for PCa TME research. Previous results suggest that increased periprostatic fat volume may be associated with shorter progression-free survival in men with prostate cancer.^1^ It has been believed that adult humans have small brown adipose tissue (BAT) depots confined to specific regions. However, recent studies have demonstrated that earlier studies have underestimated the presence of this tissue.^2,3^ Moreover, high-grade tumors are often linked to a loss of quantity of white adipose tissue (WAT), contributing to cachexia.^4^ The interactions between adipose tissue and high-risk cancer highlight the need for research on the role of adipose tissue in invasion.

## Present

With the advancement of next-generation imaging, chemical shift imaging (CSI) has emerged as an MRI technique that distinguishes between lipid and water protons within the same small voxel of space based on their differing signal intensities in in-phase and out-of-phase images. We observed a significantly higher water-to-fat ratio in periprostatic adipose tissue using CSI among high- and intermediate-risk groups compared with the low-risk group, demonstrating differences in the metabolic character of this fat depot.^5^ Immunohistochemistry staining of clinical specimens dissected during radical prostatectomy in patients with localized prostate cancer revealed the presence of brown adipocytes, marked by uncoupling protein 1 expression, in periprostatic tumor fat. These histological findings were complemented by functional in vitro assays, in which adipokines derived from periprostatic fat enhanced epithelial-mesenchymal transition and invasiveness in prostate cancer cells. Our results provide clinical and experimental evidence that the molecular and metabolic features of the tumor-adjacent adipose tissue may influence tumor progression via cytokine-mediated signaling within the TME.

## Future

Understanding the relationship between prostate cancer and adipocytes opens potential avenues for targeted therapies. Novel therapies tailored to cytokines and metabolic pathways associated with the TME hold significant promise in the era of precision medicine. Noninvasive next-generation imaging plays a crucial role in assessing the functional and compositional characteristics of the TME in the context of prostate cancer risk stratification. Future research should aim to validate imaging biomarkers across diverse cohorts and investigate TME-targeted interventions, including cytokine modulation and fat remodeling strategies. Taken together, these insights support a precision oncology approach that leverages imaging and molecular profiling to guide individualized treatment.

## References

[1] Gregg J, Surasi D, Childs A, et al. The association of periprostatic fat and grade group progression in men with localized prostate cancer on active surveillance. *J Urol*. 2021;205(1):122–8. 10.1097/Ju.0000000000001321. 32718204 10.1097/JU.0000000000001321 PMC9810079

[2] Leitner B, Huang S, Brychta R, et al. Mapping of human brown adipose tissue in lean and obese young men. *Proc Nat Acad Sci*. 2017;114(32):8649–54. 28739898 10.1073/pnas.1705287114 PMC5559032

[3] Cai Z, Zhong Q, Feng Y, et al. Non-invasive mapping of brown adipose tissue activity with magnetic resonance imaging. *Nat Metab*. 2024;6(7):1367–79. 39054361 10.1038/s42255-024-01082-z PMC11272596

[4] Álvarez-Artime A, García-Soler B, Sainz R, Mayo J. Emerging roles for browning of white adipose tissue in prostate cancer malignant behaviour. *Int J Mol Sci*. 2021;22(11):5560. 34074045 10.3390/ijms22115560 PMC8197327

[5] Shen C-Y, Pan J-K, Lin W-D, Cheng H-C, et al. Integrating magnetic resonance chemical shift imaging for localized prostate cancer risk stratification based on the impact of periprostatic brown adipocytes within tumor microenvironment. *Ann Surg Oncol*. 2025. 10.1245/S10434-025-17512-5. 10.1245/s10434-025-17512-5 PMC12317911 40437336

